# Supplementary material for: Evaluating Local Multilingual Health Care Information Environments on the Internet: A Pilot Study
Source: Int J Environ Res Public Health. 2021 Jun 25;18(13):6836. doi: 10.3390/ijerph18136836 (PMC8296914; doi:10.3390/ijerph18136836)
Supplement: Supplementary file 1 [file ijerph-18-06836-s001.zip › ijerph-1278779_Supplementary Table 1.pdf]

Supplementary Table S1. Example data extraction form

**Prefecture X**					
**Search term**	**Organization**	**Title**	**URL**	**Notes**	**Quality of translation (if suboptimal)**
**Medical system**	X Prefecture International Association	Medical information	www……jp	Links to other resources	Machine translation
	X City office	Guide for Living X City	www……jp	Typical "guide for foreigners"	
**Hospital list/ hospitals**	X Prefecture government	Health net	www……jp	Standard English search engine in Japanese page	Nice user interface
	X University Hospital	same title as organization	www……jp	No helpful information on medical care access	Some machine translation
	X Clinic	Welcome to X clinic	www……jp	Explanation of services and access	
**Emergency**	X Town government	Emergency services	www……jp	Evacuation info	
	X Tourist Information Center	If you have trouble	www……jp	General information	
**Medical interpreters**	N/A				
**National health insurance**	X City government	National Health Insurance System	www……jp	Good explanation + information on new born subsidy	
