# Supplementary material for: Evaluating Local Multilingual Health Care Information Environments on the Internet: A Pilot Study
Source: Int J Environ Res Public Health. 2021 Jun 25;18(13):6836. doi: 10.3390/ijerph18136836 (PMC8296914; doi:10.3390/ijerph18136836)
Supplement: Supplementary file 1 [file ijerph-18-06836-s001.zip › ijerph-1278779_Supplementary Table 2.pdf]

Supplementary Table S2. Demographic, geographic and medical facilities statistics for statistical analysis

Prefecture Name	Ungrouped Score Rank*	Ungrouped Informatio n Score*	Total Population[1]	Population Density[1]	Foreign Residents[2]	Overnight Tourists[3]	Number of Hospitals[4]	Number of Clinics[4]	Financial power index[5]
Japan*	-	11.9	126 443 000	565.3	2 731 093	88 589 500	8442	101 529	0.51666
Hokkaido	6.5	17.0	5 286 000	68.6	36 899	8 178 780	562	3380	0.44396
Tohoku*		8.3	8 755 000	147.9	62 520	1 392 970	612	6470	0.42544
Aomori	41.5	7.0	1 263 000	135.6	5786	379 280	96	884	0.34417
Miyagi	30	11.0	2 316 000	320.5	21 614	383 770	139	1662	0.63213
Iwate	10	15.0	1 241 000	83.8	7187	244 440	93	898	0.36522
Akita	41.5	7.0	981 000	87.9	3975	113 990	69	809	0.31081
Yamagata	47	3.0	1 090 000	120.5	7367	161 460	68	934	0.36212
Fukushima	41.5	7.0	1 864 000	138.9	14 191	152 750	128	1370	0.53818
Kantou*		13.1	43 359 000	2023.3	1 289 744	29 332 650	2034	32 601	0.79887
Ibaraki	3.5	18.0	2 877 000	478.4	66 321	213 890	178	1713	0.64915
Tochigi	38.5	8.0	1 946 000	308.1	41 648	288 460	107	1429	0.64892
Gunma	3.5	18.0	1 952 000	310.1	58 220	283 100	129	1561	0.65850
Saitama	25.5	12.0	7 330 000	1913.4	180 762	205 300	342	4225	0.76645
**Chiba**	20	13.0	6 255 000	1206.5	156 058	4 055 950	286	3778	0.77818
**Tokyo**	32.5	10.0	13 822 000	6168.7	567 789	21 766 620	651	13 184	1.19157
**Kanagawa**	20	13.0	9 177 000	3777.7	218 946	2 519 330	341	6711	0.89935
Chubu*		13.3	21 286 000	372.6	527 625	11 262 290	1196	15 765	0.55421
Niigata	35.5	9.0	2 246 000	183.1	17 285	368 130	131	1688	0.45809
Toyama	25.5	12.0	1 050 000	251.0	18 556	287 160	106	758	0.47745
Ishikawa	25.5	12.0	1 143 000	275.7	15 455	924 390	95	872	0.50666

Fukui	25.5	12.0	774 000	187.7	15 114	79 270	68	581	0.40597
Yamanashi	20	13.0	817 000	187.0	16 073	2 189 050	60	698	0.41828
Nagano	14	14.0	2 063 000	154.8	36 526	1 464 670	130	1570	0.51995
Gifu	6.5	17.0	1 997 000	191.3	55 205	1 227 320	102	1589	0.54614
Shizuoka	14	14.0	3 659 000	475.8	92 459	1 812 750	181	2711	0.72692
**Aichi**	6.5	17.0	7 537 000	1446.7	260 952	2 909 550	323	5298	0.92840
Kansai*		12.4	22 365 000	1014.9	511 739	22 494 500	1360	20 740	0.55754
Mie	32.5	10.0	1 791 000	314.5	52 087	358 420	100	1523	0.59410
Shiga	6.5	17.0	1 412 000	351.7	30 155	369 940	57	1062	0.56770
**Kyoto**	14	14.0	2 591 000	566.0	61 022	5 708 510	170	2471	0.57168
**Osaka**	2	19.0	8 813 000	4639.8	239 113	13 890 460	523	8387	0.78683
Hyogo	25.5	12.0	5 484 000	658.8	110 005	1 174 510	350	5033	0.63600
Nara	35.5	9.0	1 339 000	369.6	12 681	374 270	77	1208	0.42116
Wakayama	44.5	6.0	935 000	203.9	6676	618 390	83	1056	0.32528
Chugoku*		13.2	7 325 000	220.5	110 811	2 033 590	650	6 744	0.42606
Tottori	20	13.0	560 000	163.5	4654	183 630	44	503	0.27201
Shimane	14	14.0	680 000	103.5	9274	62 090	51	725	0.25700
Okayama	14	14.0	1 898 000	270.1	28 158	465 420	164	1661	0.52452
Hiroshima	14	14.0	2 817 000	335.4	52 134	1 212 420	244	2572	0.61866
Yamaguchi	30	11.0	1 370 000	229.8	16 591	110 030	147	1283	0.45811
Shikoku*		6.3	3 756 000	262.3	35 288	934 110	477	3393	0.37889
Tokushima	38.5	8.0	736 000	182.3	6073	111 980	113	746	0.32337
Kagawa	41.5	7.0	962 000	520.2	12 597	527 300	91	830	0.48926
Ehime	44.5	6.0	1 352 000	244.1	12 038	218 730	142	1252	0.43359
Kochi	46	4.0	706 000	102.5	4580	76 100	131	565	0.26933

Kyushu*		13.0	12 863 000	349.2	139 560	7 667 440	1481	11 453	0.40022
Fukuoka	1	20.0	5 107 000	1023.1	77 044	3 162 730	462	4 654	0.64322
Saga	35.5	9.0	819 000	341.2	6452	374 840	107	691	0.34358
Nagasaki	30	11.0	1 341 000	333.3	10 369	692 900	151	1 389	0.33570
Kumamoto	14	14.0	1 757 000	241.1	15 576	981 900	212	1 454	0.40210
Oita	25.5	12.0	1 144 000	183.9	12 951	1 339 130	157	964	0.38769
Miyazaki	35.5	9.0	1 081 000	142.7	6621	324 280	140	891	0.34349
Kagoshima	20	13.0	1 614 000	179.4	10 547	791 660	252	1 410	0.34575
Okinawa	9	16.0	1 448 000	628.4	18 025	5 250 440	94	896	0.35624

Bold faced prefectures represent Group 1 Prefectures (present in both the top 12 "foreign resident" and "tourist" lists)

* Mathematically derived (i.e. sum or arithmetic mean) information not present in source material

[1] Statistics Bureau Ministry of Internal Communications (data year: 2018) https://www.stat.go.jp/english/data/nenkan/69nenkan/1431-02.html

[2] Residents (data year: 2018) https://www.e-stat.go.jp/stat-search/files?page=1&layout=datalist&toukei=00250012&tstat=000001018034&cycle=1&year=20180&month=24101212&tclass1=000001060399

[3] Tourism (data year: 2018) https://www.mlit.go.jp/common/001274858.pdf

[4] Hospital number (data year: 2016) https://www.mhlw.go.jp/english/database/db-hh/2-2.html -

[5] Ministry of Internal Affairs and Communications (data year: 2017) https://www.soumu.go.jp/iken/ruiji/todohuken29.html

[6] Institute of Population and Social Secruity (data year: 2020) http://www.ipss.go.jp/syoushika/tohkei/Popular/P_Detail2020.asp?fname=T12-08.htm&title1=%87%5D%87U%81D%93s%93%B9%95%7B%8C%A7%95%CA%93%9D%8Cv&title2=%95%5C12%81%7C%82W+%93s%93%B9%95%7B%8C%A7%95%CA%90l%8C%FB%82%CC%95%BD%8B%CF%94N%97%EE%81C%92%86%88%CA%90%94%94N%97%EE%82%A8%82%E6%82%D1%94N%97%EE%8D%5C%91%A2%8Ew%90%94%81F2018%94N
